# Editorial: Insights in pain mechanisms 2022

**DOI:** 10.3389/fpain.2023.1290621

**Published:** 2023-09-22

**Authors:** Sascha R. A. Alles

**Affiliations:** Department of Anesthesiology and Critical Care Medicine, University of New Mexico Health Sciences Center, Albuquerque, NM, United States

**Keywords:** chronic pain, pain, neuroscience, treatment, neuroimmune

**Editorial on the Research Topic** Insights in pain mechanisms: 2022

This collection provides insights into pain and chronic pain mechanisms including pain theory, neuroimmune and glial pathways, the role of mitochondria, discussion of types of chronic pain such as neuropathic pain and arthritis, and novel treatment options.

## Pain theory

Dr. Marchand's article “Mechanisms Challenges of the Pain Phenomenon” (https://www.frontiersin.org/articles/10.3389/fpain.2020.574370/full) provides a summary of the evolution of pain theory including discussions of differences between “specificity”, “pattern”, and “gate control theory”. He also discusses the key role of diffuse noxious inhibitory controls and more recent theories such as the “pain matrix”, pain as a homeostatic emotion, interaction between endogenous inhibitory and excitatory mechanisms, and the role of genetic factors in pain phenomena.

## Neuroimmune and glial mechanisms

This collection includes a review that discusses the role of neuroimmune and glial mechanisms in arthritic pain from Dr. Corr and colleagues (https://www.frontiersin.org/articles/10.3389/fpain.2022.1018800/full). In it they discuss the role of macrophages and glial cells in the peripheral joint in arthritis such as rheumatoid arthritis (RA), one of the most well studied types of inflammatory arthritis. These non-neuronal cells play a key role in the onset and maintenance of arthritis-associated pain. Several mouse and rat models of arthritis are summarized including mono-articular inflammation induced by Complete Freund's Adjuvant (CFA) injection into the knee or ankle. This review also discusses signaling in the dorsal root ganglia (DRG) involving cytokines, macrophages, satellite glia, microglia and astrocytes. The concept of the “arthritic brain” is also mentioned to draw attention to the relevance of co-morbidities such as depression and anxiety in patients with RA. Finally, potential treatment options are presented that target the peripheral and central nervous system.

## The role of mitochondria in pain

Dr. Eijkelkamp's group provide a summary of the latest findings on the role of mitochondria in inflammatory and neuropathic pain in a detailed review (https://www.frontiersin.org/articles/10.3389/fpain.2022.1013577/full). They discuss how dysfunction of the mitochondria can connect inflammation to neuronal sensitization and subsequent pain. Mitochondria play an essential role in regulating inflammatory responses, but they also regulate action potential firing and neurotransmitter release. The high energetic demand of neurons means that mitochondrial dysfunction can wreak particular havoc on them. Key aspects of how mitochondrial dysfunction can contribute to pain-related processes are discussed in terms of effects on mitochondrial respiration, oxidative stress, Ca^2+^ buffering, inflammasome activation, quality control mechanisms including mitophagy, mitochondrial biogenesis, fusion and fission, and also, transport mechanisms. The review also discusses how mitochondrial dysfunction can affect non-neuronal cells in rheumatic diseases. Promising approaches for correcting mitochondrial dysfunction as potential treatments for pain are mentioned. Concluding remarks from this review call for more studies on female mice, the role of age and primary human DRG with regards to mitochondrial dysfunction in pain.

## Neuropathic pain

Dr. Smith provides a review on neuropathic pain, a type of chronic pain resulting from injury to or disease of the nervous system (https://www.frontiersin.org/articles/10.3389/fpain.2023.1220034/abstract). This often intractable form of chronic pain involves neuronal, glial and immune cell types. The review also includes discussion of higher brain centers such as the thalamus, cingulate and sensory cortices. Dr. Smtih also points out that in spite of numerous studies that the field has yet to advance to the clinic any major therapeutic breakthroughs. Potential reasons for this are described as being due to use of only male rodents in studies, differences in pain processing between rodents and humans and lack of stratification of human pain phenotypes in clinical studies. Future studies should take these factors into account.

## Non-pharmacologic approaches for pain treatment

In the only research article in this collection, Dr. Sivan presents data showing that acute fasting reduces pain tolerance in healthy subjects (https://www.frontiersin.org/articles/10.3389/fpain.2023.1153107/abstract). Since food consumption has been linked to analgesia, his group decided to investigate this relationship in human subjects. A total of 25 participants were recruited and subject to non-fasting or 12 h-fasting sessions. Pain was evaluated using the cold pressor test (CPT). Pain tolerance was assessed using a Stroop task in both attentive and distracted states. In addition, data on mood and hunger was captured using the Profile of Mood States (POMS) and a 10-point hunger scale. Results showed that acute fasting reduced pain tolerance in healthy subjects and that effects were independent of gender, attention or mood. Further studies such as this in subjects with chronic pain would be helpful to explore fasting as a non-pharmacologic treatment option.

